# Adaptations in the transformation of cassava (*Manihot esculenta* Crantz; Euphorbiaceae) for consumption in the dietary management of diabetes: the case of Palikur, or Parikwene People, from French Guiana

**DOI:** 10.3389/fnut.2023.1061611

**Published:** 2023-05-12

**Authors:** Michael Rapinski, Alain Cuerrier, Damien Davy

**Affiliations:** ^1^Institut de Recherche en Biologie Végétale (IRBV), Université de Montréal, Jardin botanique de Montréal, Montréal, QC, Canada; ^2^UAR 3456 Laboratoire Ecologie, Evolution, Interactions des Systèmes Amazoniens (LEEISA), CNRS, Université de Guyane, IFREMER, Cayenne, French Guiana

**Keywords:** diabetes – quality of life, Palikur, French Guiana, cassava (*Manihot esculenta* Crantz), nutrition therapy, operational sequence, ethnomedicine in T2DM, cultural keystone species

## Abstract

**Introduction:**

In the French overseas department of French Guiana, in South America, nutrition therapy for the management of diabetes is based on French guidelines. However, this region is demographically diverse and includes several populations of Indigenous Peoples, Parikwene among others, also called Palikur. Due to socio-economical, cultural, and geographical differences, along with distinctions in the local food system, dietary recommendations, which many consider in the context of post-colonial power dynamics, are not well suited to local populations. In the absence of suitable recommendations, it is hypothesized that local populations will adapt their dietary practices considering diabetes as an emerging health problem.

**Methods:**

Seventy-five interviews were conducted with community members and Elders, as well as healthcare professionals and administrators providing services to the Parikwene population of Macouria and Saint-Georges de l’Oyapock communes. Data regarding the representation of cassava (*Manihot esculenta Crantz*) consumption and diabetes were collected via semi-structured interviews and participant observation (i.e., observation and participation in community activities), namely via participating in activities related to the transformation of cassava tubers at swidden and fallow fields.

**Results and Discussion:**

Parikwene have adapted the transformation of cassava tubers for their consumption in the management of diabetes.The importance of cassava tubers as a staple and core food to the Parikwene food system was established by identifying it as a cultural keystone species. Narratives illustrated conflicting perceptions regarding the implication of cassava consumption in the development of diabetes. Adaptations to the operational sequence involved in the transformation of cassava tubers led to the production of distinct cassava roasted semolina (i.e., couac), based on organoleptic properties (i.e., sweet, and acidic couac). Preferences for the consumption of acidic couac were grounded in the Parikwene knowledge system, as well as attention to diabetes related symptoms and glucometer readings.

**Conclusion:**

These results provide important insights related to knowledge, attitudes, and practices in developing locally and culturally adapted approaches to providing dietary recommendations in the treatment of diabetes.

## 1. Introduction

Type 2 diabetes (T2D) can be described as a metabolic disorder characterized primarily by insulin resistance and impaired glucose uptake that consequently leads to hyperglycemia (1). In combination with pharmacotherapy, nutrition therapy along with nutritional advice and information are considered essential for the effective treatment and management of diabetes (type 1 and 2) and its prevention for those at risk of developing T2D (2–6). Indeed, lifestyle changes, including sedentarization and changes in dietary patterns (i.e., food and nutrition transition), are among several factors implicated in the worldwide increase of diabetes (7, 8). This is particularly true among populations with a history of colonialism that has considerably accelerated the rate of changes in the way of life by undermining family values, and societal structures as well as cultural and spiritual practices (9–13).

**Figure 1:**
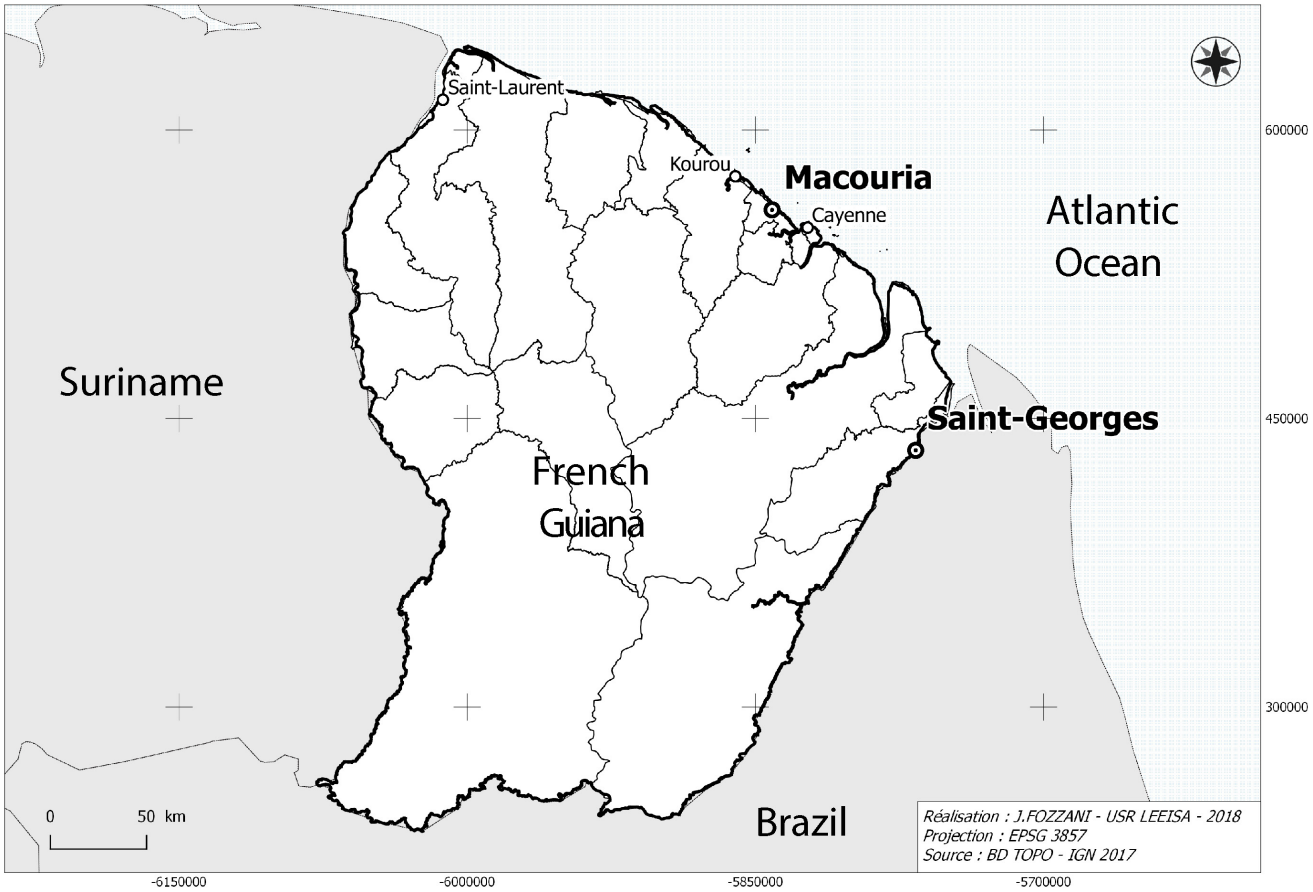
Map showing the location of Parikwene villages in French Guiana, namely in the communes of Macouria (5°00′N, 52°28′W) and Saint-Georges de l’Oyapock (3°53′N, 51°48′W). Cartography: Jérôme Fozzani—USR LEEISA. Projection in pseudo-Mercateur.

In comparison to France, the prevalence of diabetes is notably higher in French overseas departments (14). That is particularly true in French Guiana, where 8.08% of the population, age-adjusted, was estimated to be diabetic in 2015 as opposed to 5% of the population in France, rates that have roughly doubled in a 10-year span (10–12, 15, 16). The demographic composition of French Guiana includes a large number of diverse Creole, Maroon, and Indigenous cultural groups characterized by considerable movement and migration from Haiti and neighboring countries (17), namely Suriname and Brazil (Figure 1). As elsewhere on the American continents where Afro-Descendants and Indigenous Peoples were subject to colonization, these populations are considered among those with the highest risk of developing diabetes (13, 18–24).

**Figure 2:**
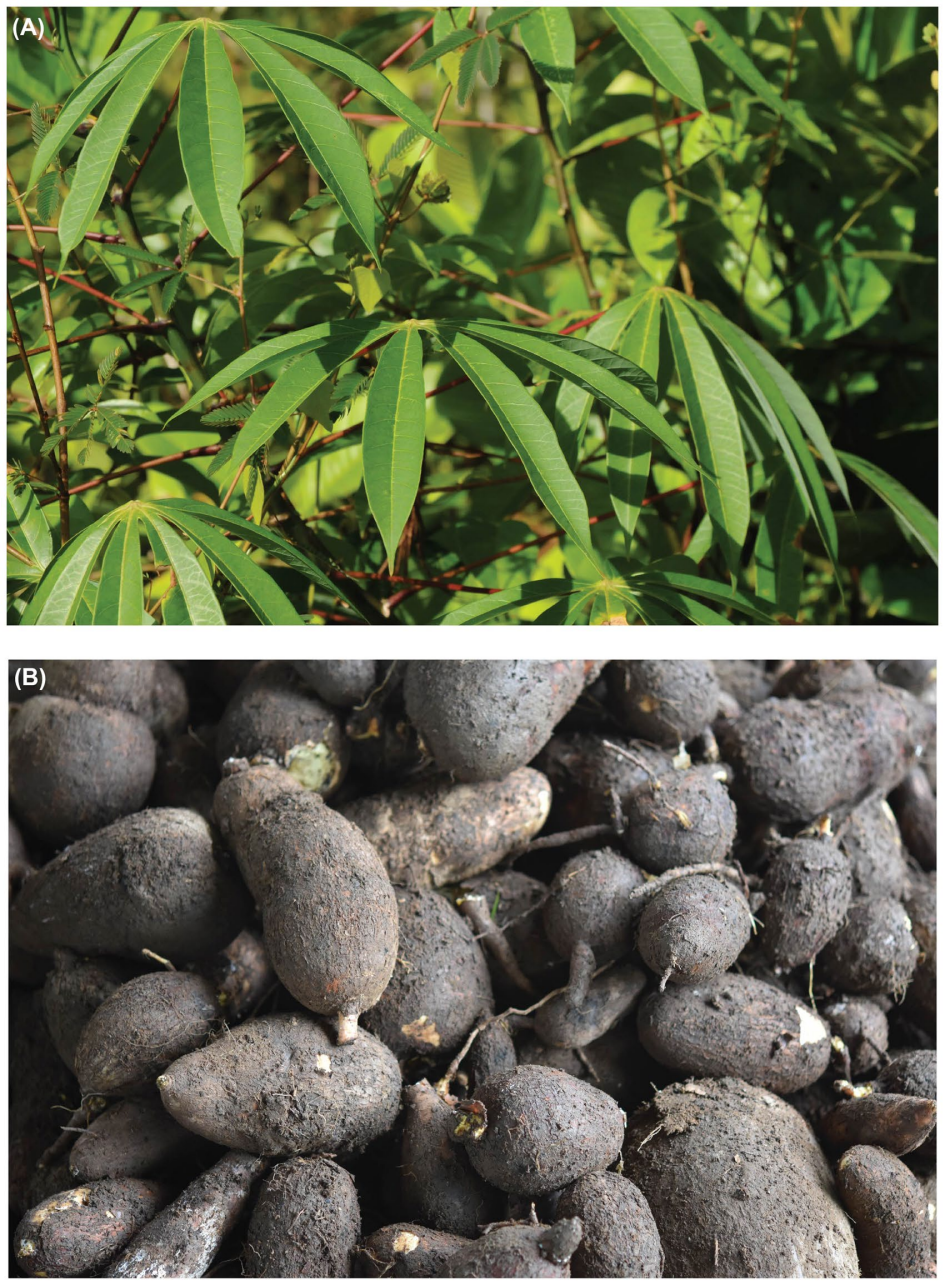
**(A)** Aerial parts and **(B)** tubers of bitter cassava (*Manihot esculenta*). Photo credits: A. Cuerrier **(A)**, M. Rapinski **(B)**.

Parikwene are a cross-border Indigenous People living in French Guiana and Brazil. Despite relatively good health status from 1925 to 1950 (25, 26), the first reports of diabetes were recorded among those living in Brazil by 1978 (27). Although Parikwene have been in contact with European societies since the 16th century, the speed of lifestyle changes has accelerated since the mid-1960s (28), especially since 2003 with the opening of the region along the Brazilian border through road access (29–32). Such lifestyle changes have led to a growing reliance on store-bought and manufactured foods (i.e., powdered milk, coffee, condiments, oil, cookies, rice, butter, bread, and soda) to the detriment of food production activities such as hunting, fishing, and swidden-fallow cultivation of cassava (*Manihot esculenta* Crantz; Euphorbiaceae; Figure 2), which remains a staple of the diet nonetheless (29, 32–34). Nowadays, Delocalized Centers for Prevention and Care (*Centres Délocalisés de Prévention et de Soins*) of the Andrée Rosemon Cayenne Hospital Center (*Centre Hospitalier de Cayenne Andrée Rosemon*) record the largest number of treated diabetic patients in Saint-Georges de l’Oyapock (35), home to a sizeable Parikwene population on the French Guianese side of the Brazilian border.

In keeping with the relevance of nutritional therapy for the treatment of T2D, several organizations, such as the World Health Organization (36), the American Diabetes Association (37), Diabetes UK (2), the French Haute Autorité de Santé (38) and Diabetes Canada (3), have provided support for the distinction of high and low glycemic indices (GIs) of food in providing nutritional and dietary recommendations. Examples of food items with a high GI include those with a high starch content derived from grains like wheat (*Triticum aestivum* L.; Poaceae) and rice (*Oryza sativa* L.; Poaceae), as well as root vegetables like potatoes (*Solanum tuberosum* L.; Solanaceae), yams (*Dioscorea* spp.; Dioscoreaceae) and also cassava (39). According to the USDA (40), 100 g of raw cassava contains 60 g of water and 38 g total carbohydrate, which represents 12% of the daily value. Carbohydrates are therefore the main macronutrients and sources of energy followed by proteins (1.4 g), fat (0.3 g) and various micronutrients, like vitamins C and B6, and magnesium (40).

Guidelines for the treatment of diabetes in French Guiana are generally applied from European and French recommendations (14, 38, 41, 42). Arguably, these standards are not suitably adapted for the French Guianese context (41), and the latest French National Health Nutrition Program (*Programme national nutrition santé 2019–2023—*PNNS 4) has declared the necessity of adapting their program to the specificities of overseas territories (42, 43). Whereas many healthcare professionals that include diabetologists and nutritionists are trained in France, the dietary profile of French Guiana’s diverse population is noticeably different to the European territory of France and calls for locally adapted approaches to dietary interventions (14). We hypothesize that in the absence of suitable dietary recommendations, local populations will nonetheless adapt their dietary practices considering diabetes as an emerging health problem. Working with French Guiana’s Parikwene People, perceptions and representations of consumption of cassava were documented, along with grassroot strategies and practices developed for the dietary management of diabetes.

## 2. Materials and methods

### 2.1. Ethnographic background

Commonly referred to as Palikur in French and English, the Parikwene, or Pahikwene, who self-identify as such, are a group of Peoples that speak Parikwaki, a language within the Maipuran sub-family of the Arawak language family (29). During the 19th century, the majority of Parikwene inhabited the banks of the Rio Urucauá, now located in the Amapá state of Brazil, though oral history points to a small presence on both sides of the Oyapock River that now makes the border between France and Brazil (44). Migration into their current settlements in French Guiana began after the region corresponding to the state of Amapá was conceded to Brazil in 1900. However, major migrations are traced back to the 1960s with the creation of the village of Espérance I, in Saint-Georges de l’Oyapock (31), and later on with the village of Kamuyene in Macouria. Due to French legislation preventing the recording of census data distinguishing ethnicity, there are no precise numbers on the Parikwene population in French Guiana, nor on their prevalence of illnesses like diabetes. Self-reported numbers from Parikwene leaders in Saint-Georges de l’Oyapock and Macouria bring the estimates in these communes closer to 1800 Parikwene in French Guiana in 2018, doubling their estimated numbers from 850 people in 2001 (29).

Included in this study are the villages of Kamuyene and Norino in the commune of Macouria, as well as the villages of Espérance I and Espérance II in the commune of Saint-Georges de l’Oyapock (Figure 1). Due to their size and proximity from one another, inhabitants of each village are referred to in association with their commune (i.e., Parikwene of Macouria and Parikwene of Saint-Georges de l’Oyapock). Vehicular languages in these villages include Parikwaki, French Guianese Creole, French and Portuguese.

### 2.2. Consultations

Fieldwork took place from June 2017 to July 2018. Altogether, 75 interviews were completed with 101 participants with a sex ratio (men/women) of 0.87. The breakdown of interviews is presented in Table 1. Exchanges were carried out in the interviewees’ languages of preference, namely French and French Guianese Creole, as well as Parikwaki with the help of an interpreter. Participants were selected based on their association to one of four demographic categories and were first identified by snowball sampling. Names were initially provided through existing relationships between community members and one of the authors (DD) who has been working with Parikwene communities for over 20 years (33, 45–47), and by consulting with local *Chefs coutumiers* (i.e., village Chiefs).

**Table 1:** Interview breakdown by communities and description of participant categories.

**Community**	**# of interviews**	**# of participants**	**Mean age (±stdev)**
Saint-Georges	32	43	53.4 (±15.8)
Macouria	28	42	51.1 (±15.4)
Cayenne^a^	15	16	42.3 (±10.6)
Total	75	101	50.4 (±15.2)
**Category**		**Example**	
Community members and healthcare users			
Knowledge holders		Elders, healers	
Healthcare users		All other community members	
Healthcare network staff			
Healthcare administrators		Directors, administrators	
Healthcare professionals		Doctors, nurses, nutritionists	

a Interviews conducted with participants working in or with the healthcare sector.

### 2.3. Data collection

Data were collected via semi-structured interviews and participant observation (i.e., observation and participation in community activities), namely via participating in activities related to the transformation of cassava tubers at swidden and fallow fields and observing each step of the operational sequence, or *chaîne opératoire*. This study was conducted within the scope of a wider research project whereby interview questions related to knowledge, attitudes and practices pertaining to diabetes and its treatment. Nomenclature and terminology were recorded in Parikwaki. Because various writing systems have been developed over time, preference was given to the system developed by Green et al. (48), preferred by participants from Macouria and Saint-Georges. Validation and confirmation of terminology, nomenclature and various other associated concepts were conducted during two subsequent fieldtrips to the villages in 2019 and 2022.

### 2.4. Ethics statement

Informed consent was obtained from participants prior to each interview. Consent forms and questionnaires were approved by each village’s *Chef coutumier*, along with the Ethics Committee of the Université of Montréal (Certificate #: CERAS-2016-17-081-P). Overall, the working approach was conducted with respect to the International Society of Ethnobiology’s Code of Ethics (49). This study was not constrained by France’s ratification of the Nagoya protocol as cassava production, topping 302 million tonnes worldwide (50), is consequently well known to human populations at large and the scientific community.

### 2.5. Data analyses

All interviews were transcribed verbatim. Transcripts were then stored and handled with NVivo qualitative data analysis software (51) to identify, manage, link, and retrieve coded data. Thematic analysis and sequential focused coding centered on the cultivation, transformation and consumption of cassava, as well as narratives regarding its connection with diabetes. Results from thematic analyses distinguishing cassava products and their perceived impact on diabetes can be found in a supplementary data file. Verbatim quotes translated into English by the authors are used to illustrate narratives.

## 3. Results and discussion

### 3.1. Cassava as a cultural keystone species

Early on (3,000–4,000 B.P.), Proto-Arawak Peoples, to which Parikwene are linguistically related, are believed to have relied on domesticated cassava as an important food source, thus leading to the development of numerous Arawakan societies whose food systems have and continue to rely on cassava agriculture (52). To understand the role of cassava in the contemporary Parikwene food system, as well as narratives associated with its consumption in relation to diabetes, it is helpful to consider this species as one of exceptional significance to Parikwene, i.e., a cultural keystone species (53, 54). To assess the cultural significance of a species, Garibaldi and Turner (54, p. 5) propose six indicators, which are addressed herein primarily through a dietary lens: “(1) intensity, type, and multiplicity of use; (2) naming and terminology in a language, including the use as seasonal or phenological indicators; (3) role in narratives, ceremonies, or symbolism; (4) persistence and memory of use in relationship to cultural change; (5) level of unique position in culture, e.g., it is difficult to replace with other available native species; and (6) extent to which it provides opportunities for resource acquisition from beyond the territory.”

#### 3.1.1. Varieties

“Naming and terminology in a language” is best assessed by considering the recognition of varieties and their names incorporated in the Parikwaki language (Table 2). Parikwene distinguished varieties of bitter cassava (*kaneg*) from sweet cassava (*awava*). This differentiation is crucial as the content of cyanogenic compounds discerns the former from the latter; elevated concentrations of these toxins in bitter cassava varieties require elaborate transformation procedures to avoid cyanide toxicity (55, 56). Furthermore, specific varieties recognized locally by Parikwene were also given specific names. Although making a full inventory of these varieties was outside the scope of this study, the chief of Espérance 1 reportedly identified nearly 42 varieties of which four were explicitly cited by participants during this study, namely *kaneg sansan*, *kaneg kalisha,* *kaneg wauviye*, and *kaneg burink*. Past studies have inventoried up to 29 varieties of cassava tubers named by Parikwene, namely two sweet and 27 bitter varieties (31, 57). In 2002, there were 14 varieties that were still being cultivated (57). Furthermore, Parikwene classified bitter cassava varieties within two major categories: (i) white and (ii) yellow.

**Table 2:** Parikwene nomenclature of *Manihot esculenta* and terms given to foodstuff derived from its transformation.

Parikwaki	French Guianese Creole	English
Nomenclature of *M. esculenta*		
Kiniki	Plan mangnok	Cassava plant (above ground)
Awava	Kranmangnok	Sweet cassava tuber (underground)
Kaneg	Mangnok	Bitter cassava tuber (underground)
Kaneg sansan	Mangnok kalité blan	Variety of white bitter cassava
Kaneg kalisha	Mangnok kalité jonn	Variety of yellow bitter cassava
Kaneg wauviye	Mangnok kalité jonn	Variety of yellow bitter cassava
Kaneg burink	Mangnok kalité jonn	Variety of yellow bitter cassava
Foodstuff derived from bitter cassava tubers		
Kayut	Mousach	Starch
Kayut kuwagaki	Tapioka	Tapioca (dry)
Kayut igiye	Lanpwa	Starch (wet)
Karahu	Kwabyo	Cassava juice (tucupi)
Wat marehpeket (bugut)	Kasav	Cassava flatbread
Puveye	Kwak	Couac
Dishes and beverages prepared from bitter cassava tubers		
Tukuiska (bugurak)	Chibé	Chibè (beverage of couac soaked in water)
Matit puveye	Matété kwak	Mash or cream of couac (dish)
Kusimna	Takaka	Tacacá (dish prepared from tapioca)
Wonska	Kachiri	Cassava beer

#### 3.1.2. Dietary staple

**Figure 3:**
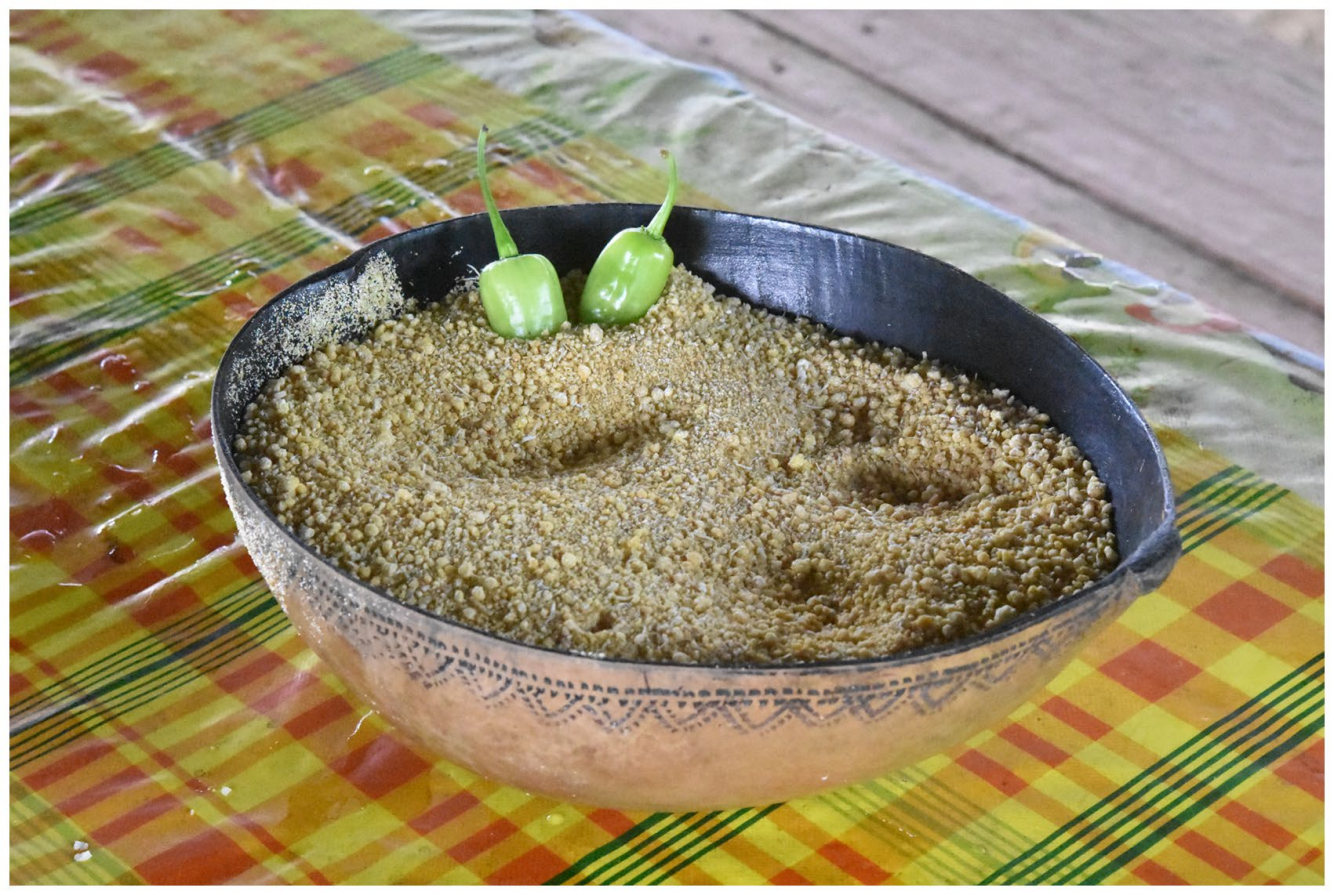
Torrefied semolina made from bitter cassava tubers, called *kwak* in French Guianese Creole and *puveye* in Parikwaki, served with hot peppers, or *atit*. Photo credit: M. Rapinski.

**Figure 4:**
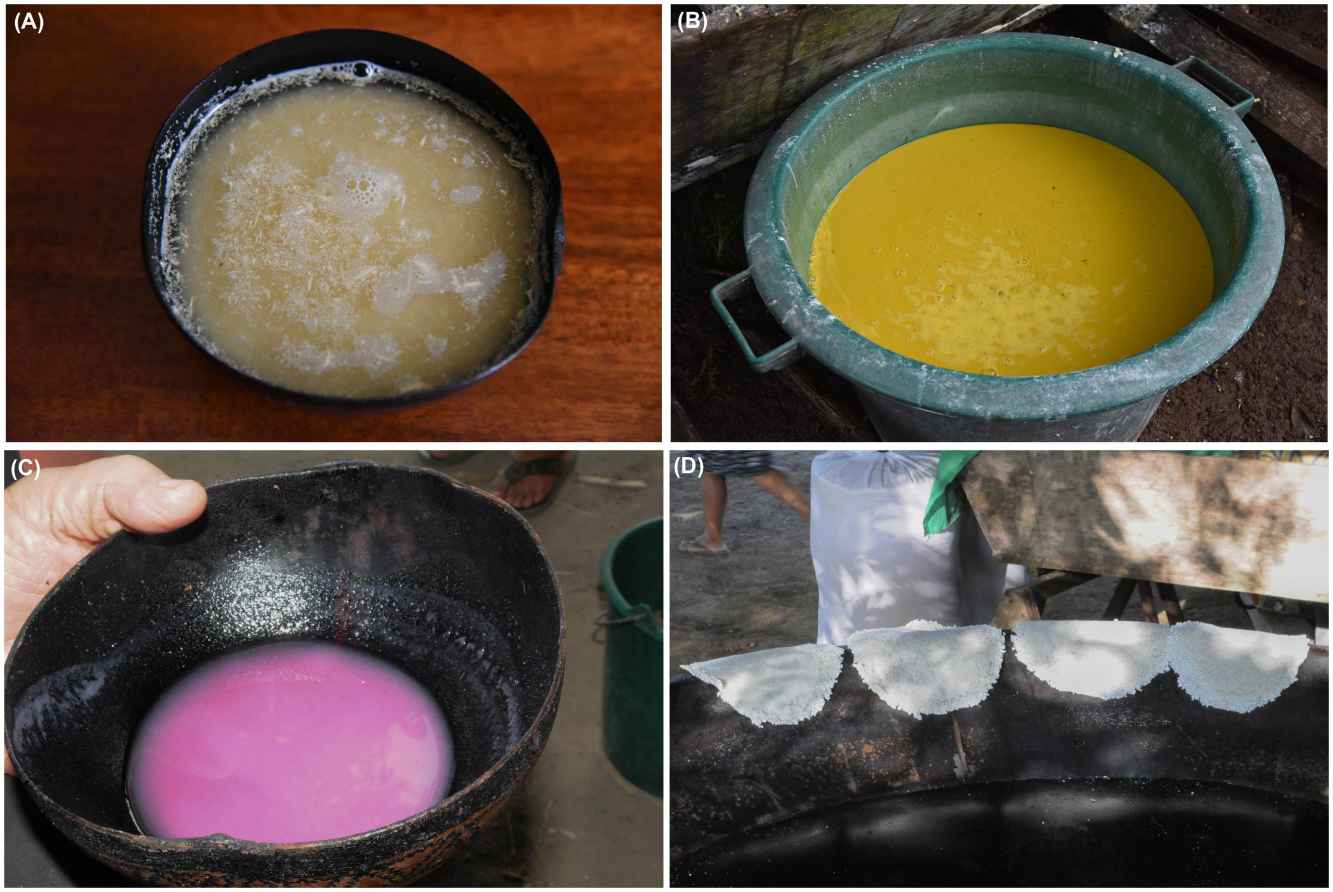
**(A)** Chibé, called *bugurak* in Parikiwaki, a thirst-quenching beverage made from couac soaking in water. **(B)** Cassava juice decanting at a “cassava house,” near a swidden and fallow field, to separate the starch that will go in the fabrication of tapioca (*kayut*) from the liquid that will serve as a culinary condiment (*karahu*). **(C)** Cassava beer, called *kachiri* in French Guianese Creole and *wonksa* in Parikwaki, made from cassava tubers fermented with sweet potatoes. **(D)** Cassava flatbread, called *wat marehpeket* in Parikwaki, made from cassava starch, or *kayut* in Parikwaki. Photo credits: A. Cuerrier **(A, C, D)**, M. Rapinski **(B)**.

“Intensity, type and multiplicity of use” is assessed here by considering the diversity of dietary uses attributed to cassava. Sweet cassava tubers, such as *awava*, were consumed, namely boiled, in a similar fashion to several starchy root vegetables that accompany meals like dasheen, or taro (*Colocasia esculenta* (L.) Schott; Araceae), sweet potatoes (*Ipomoea batatas* (L.) Lam.; Convolvulaceae) and various species of yam (*Dioscorea* spp.; Dioscoreaceae). However, bitter cassava tubers were more versatile as they were the main source of several derived foodstuff (Table 2). The most popular food item derived from bitter cassava was a torrefied semolina locally called *kwak* in French Guianese Creole or *puveye* in Parikwaki (Figure 3). Couac was present in the household of every Parikwene participant interviewed and could be consumed at every meal, as well as in-between mealtime snacks in the following ways:

- added to hot breakfast beverages, like coffee or hot chocolate;
- added to bowls of palm nectars like bacaba, or *woki* (*Oenocarpus bacaba* Mart.; Arecaceae), patawa, or *pataw* (*Oenocarpus bataua* Mart.; Arecaceae) and açaí, or *was* (*Euterpe oleracea* Mart.; Arecaceae);
- as an accompaniment to main meals, such as grilled or boiled meats and fish, whereby couac is either added to an individual’s plate, or meat and fish are dipped in a communal platter of couac;
- as a dipping condiment for fruits, like mangoes (*Mangifera indica* L.; Anacardiaceae) and pineapple (*Ananas comosus* (L.) Merr.; Bromeliaceae);
- soaked in fresh water to make a thirst-quenching beverage called *bugurak*, or chibé (Figure 4A);
- boiled to make a cream or mash akin to porridge called *matit*.

Starch (*kayut*) extracted from bitter cassava tubers and transformed into tapioca (*kayut kuwagaki*) was also used as an additive to hot beverages and bowls of palm nectars, but also in the preparation of specific dishes like *kusimna*, or tacacá. This dish is as varied as the choice of broth, including the liquid from which starch is extracted (*karahu*), which is thickened by tapioca and used in its preparation. Furthermore, cassava juice (*karahu*) could also be used to cook fish or meat (Figure 4B), and is commonly turned into a spicy condiment (*atit karahu*) by adding hot peppers, *atit* (*Capsicum annuum* L.; Solanaceae). Finally, cassava flatbreads (*wat marehpeket*) and cassava beer (*wonska*) were also consumed occasionally or on special occasions (Figures 4C,D). Though cassava beer formerly had a real importance in daily life, particularly during *sinal* ceremonies (25, 58, 59), the past few decades have seen the influence of evangelical churches that have forbidden its consumption (60, 61). Nonetheless, the consumption of cassava among Parikwene falls within the scope of food systems of Peoples from the Amazon basin and the Guiana Shield (56, 62–64), whereby tubers remain a dietary staple (31), which is best described as a key asset in the fight for food sovereignty.

#### 3.1.3. Lifestyle pillar

Cassava’s “level of unique position in culture” is best understood in relation with swidden and fallow fields as a place of high biocultural salience, i.e., a cultural keystone place (65). Together, they form structuring pillars of Parikwene lifestyle and land-use activities. Although swidden and fallow agriculture centers primarily around the cultivation of bitter cassava tubers, which is intricately timed with the seasons (66–68), several dietary plants are also cultivated and harvested on the periphery of fields, which are increasingly and predominantly monospecific (31), and particularly near shelters. These include edible palms like patawa, bacaba and açaí, fruits like mangoes, cashews (*Anacardium occidentale* L.; Anacardiaceae), papayas (*Carica papaya* L.; Caricaceae), bananas (*Musa paradisiaca* L.; Musaceae), pineapples, and several citruses (*Citrus* spp.; Rutaceae), starchy root vegetables, like dasheen, sweet potatoes and yams, as well as several medicinal plants (69, 70). Moreover, swidden and fallow fields act as an access point to secondary and primary forests that provide a number of resources, acquired through wild harvesting, hunting and fishing, which can increase dietary diversity, but also be used for medicinal and technological purposes. Finally, the transformation of cassava tubers typically occurs at specifically designed shelters near the fields called “*payt hihevinwa*,” or “cassava house” (33), and shape the development of specialized tools employed at different stages of processing cassava into various foodstuff, namely couac, tapioca, and cassava juice. Such tools include sieves and cassava squeezers handcrafted from wild sourced material and steeped in a highly developed skill set and knowledge system related to basketry (45, 46). The extent to which cassava can hardly be replaced by another species in both spawning material culture and acting as a staple and core food item of Parikwene dietary systems is highlighted by the remaining indicators of a cultural keystone species discussed hereafter.

Although the use of cassava squeezers in detoxifying cassava tubers has mostly been replaced by mechanical presses, their fabrication remains relevant to a small-scale artisanal market economy (31, 45), which consequently “provides opportunity for resource acquisition from beyond the territory.” This also applies to couac, which is purchased and supplied directly to Parikwene villages in Macouria through a distribution network beginning in Saint-Georges, and is eventually sold on local and departmental markets (31), where it is highly reputed. Despite the number of people growing cassava for couac production may be diminishing overall (30, 71), there is an apparent specification of roles in Parikwene society through the formation of semi-professional cassava farmers, a process that had already begun by the turn of the millennium (31). This process is akin to what has been observed among Parikwene hunters and their hunting activities (61), or activities concerning basketry (45, 47). Despite barriers to accessing cultivable lands (71), local farmers’ associations like Wacapou facilitate access to a larger market economy by enabling the professionalization of agricultural activities and by providing the means and resources to tend large cassava fields and mechanize production with new tools.

Finally, activities related to harvesting and transforming cassava tubers are structured around collective events that solicit mutual help among families and the participation of several generations of family members. *In fine,* swidden and fallow cultivation of cassava acts as a nexus for subsistence activities (i.e., dietary, medicinal, technical, and economical) and strengthening social relations that ensure cultural and linguistic continuity. This maintains the species ubiquity in the collective cultural consciousness that ensures “persistence and memory of use in relationship to cultural change.” This relationship to change is tightly woven into cassava’s “role in narratives, ceremonies, or symbolisms.” Although these can be addressed through various angles, such as the place and role of *wonska*, or cassava beer in ceremonies, it is through its prominently featured place in narratives regarding health and diabetes and consequent adaptations to the operational sequence of couac production that it is illustrated hereinafter.

### 3.2. Cassava and diabetes

“If we drop couac, we cannot eat. The problem is that we are going to die.” LS6022, Macouria.

Due to the high starch content of cassava tubers, their consumption is featured in narratives provided by healthcare professionals. When asked about the factors responsible with the development of diabetes, cassava consumption was cited by 79.3% of participants linked to the healthcare sector. Some doctors in Cayenne cited the effect of cassava toxicity on pancreatic injury, LS4013 explains: “Couac is made from bitter cassava which must be washed well because otherwise it is toxic, especially for the pancreas.” Couac was not cited as a direct cause of diabetes, but one of many intertwined factors that involve transformations to Parikwene food systems (e.g., transitions to market food and increased consumption of sweet and fat foods and beverages) and lifestyle (e.g., reduced physical activities). However, one doctor from Macouria explains some of the difficulty related to language barriers when discussing dietary recommendations around couac:

But actually, couac is part of… the glycemic indices which are still quite high as starchy foods […] Doctors, I think they take shortcuts, sometimes, when they explain to people who don't speak French very well, and so on. So, sometimes, they will tell them: 'No, no, but couac, ok, it's not worth it. Take that off [your plate], yeah.' LS6002, Macouria.

Increasing with the ripeness of fruits and the cooking time of starchy tubers and grains, the GIs of a food can vary greatly according to a wide range of factors, such as species variety (39). Moreover, the GIs of common staple foods (e.g., rice and wheat) depend very much on a number of processing, refining and cooking practices (39). Despite being one of the five largest staple crops cultivated worldwide (72), cassava seems relatively underrepresented and overlooked in the International Tables of GI, where it appears as a boiled root (39). Although these tables focus on a number of commercially available and transformed food products, fruits and starchy foods available worldwide, caution is advised when interpreting GIs with few data points (39). Notwithstanding their shortcomings, GIs can be useful in guiding dietary choices; in the absence of data for a specific food item, information is extrapolated to assign low GIs to vegetables and high GIs to flour products (39). Although foods and food items specific to certain regions are included, knowledge of local foods is required (39). Hence, a great deal of work is needed to make these tables fully comprehensive, this is particularly true for the purpose of providing meaningful dietary recommendations regarding cassava consumption.

Because nutritional and dietary advice and information is provided during visits and interactions with doctors and nurses, such information has naturally reverberated in Parikwene responses and explanations regarding the development and progression of diabetes. However, what is resoundingly clear is an overwhelming interpretation that healthcare professionals are blaming the consumption of couac in the development of diabetes. One resident from Macouria, LS6026, explains that “a lot of doctors say it’s because [Parikwene] Indians eat a lot of couac and they find diabetes. And then, they catch diabetes in the couac.” Considering the cultural importance of this dietary staple, some find it difficult to believe the perceived rhetoric, as exemplified by one resident from Saint-Georges, LS5008, who vehemently opposed the idea: “And then the doctors, they talked about not eating couac. It’s couac that has… ‘It’s couac that caused diabetes.’ It’s not couac! They said… Doctors, they said: ‘It’s couac that causes diabetes.’ But that’s not true, it’s not couac.”

Some residents interviewed do not necessarily deny the evidence regarding the carbohydrate content of couac. When rationalizing the development of diabetes within the context of the nutrition and food transition experienced by Parikwene People, it is the approach taken by doctors and the simplicity of their messaging that is put in question, as one resident from Saint-Georges explains:

That’s what they put in their heads. It’s the doctors, when they do campaigns, who say that… Well, it’s true, they [the research community] did research on couac and they said: ‘Couac has a lot of sugar.’ So, they [the doctors] say: ‘Couac gives diabetes.’ It’s the doctor when he campaigns, he prevents. That’s why they put it in their [Parikwene] head, they say that it’s couac that gives diabetes. For me, well, that’s not true. For me, it does not make any sense, it is not true… It is not true. It would be years back, and they are not diabetic. How did they [the Elders]… they died at 100 years, 90 years old, and they did not even have diabetes. Now, why now us, the descendants now, not even at 15, at 20, are we diabetic? For what reason? Why? Because we do not just eat couac. We consume everything that is not good for the body. LS5012, Saint-Georges.

Another participant interviewed dismisses the centrality of couac by echoing narratives on the nutrition and food transition and recentering diabetes as a global phenomenon:

I spoke with a doctor at the center and he said to me: ‘Yeah, but why among the Palikur Amerindian, there are a lot of… There are many diabetics? Because… Is it because of your food and all that?’ I said: ‘No.’ Because, well… Our elders, before, they have…well, they rarely had diabetes. Because they have eaten… They are not eating… I say: “Well, now, in relation [to the past], we use products… We eat your products, your food, and all that. Why, in mainland France, there are people, they are diabetics? And the United States… In the United States, well, they do not eat couac. And why are they diabetic?’ LS5010, Saint-Georges.

From the first cases of diabetes recorded among Parikwene living in Brazil during the 1970s (27), food acculturation has been pointed out as a culprit, with some authors also suggesting the high consumption of cassava tubers (27, 73). Since the late 1960s, various forms of diabetes (i.e., J type and pancreatic diabetes) have been associated with specific dietary practices, and more specifically cyanide-yielding substances, such as cassava (74, 75). Geevarghese (76) explains this is the result of cassava’s high carbohydrate content and the presence of cyanogenic glycosides inducing pancreatic injury. The suggestion that chronic cyanide toxicity due to the ingestion of cassava tubers is one of the possible etiological factors leading to pancreatic injury is contentious as a case–control study found no association between its ingestion and tropical pancreatitis (77). Furthermore, several studies have failed in establishing a direct link between cassava consumption and diabetes since the late 1980s. An *in vivo* study found that the long-term ingestion of cassava did not produce diabetes or pancreatitis in rats after 1 year of feeding (78). Another *in vivo* study found that cassava-enriched diets were not diabetogenic in rats, but rather aggravated hyperglycemia in diabetic rats (79). A clinical study failed to find evidence that chronic consumption of cassava flour containing cyanide predisposes to diabetes mellitus (80). Another study did not find that high dietary cyanide exposure, or cassava toxicity, had significant effect on the prevalence of malnutrition-related diabetes mellitus (81).

These studies do not undermine the mounting evidence regarding the neurotoxicity of cyanogenic compounds (82–84). Although such compounds, such as linamarin and lotaustralin can trigger neurological disorders like tropical ataxic neuropathy (82, 84), the link between these neurotoxins and diabetic neuropathy deserves further research. Nonetheless, the toxicity of cassava varies widely between varieties. Not only is their content in cyanogenic glycosides variable, but their remnant content also depends on the use of several processing techniques that lead to their elimination like grating, soaking, pressing, fermenting and heating (56, 85). Fermentation alone can lead to reductions in cyanide by 70–95%, whereas combined processing methods (i.e., soaking, fermentation and roasting) can leave products nearly free from cyanogens (55, 86, 87).

### 3.3. Dietary adaptations

It’s normal for the doctor to say that couac is not good for diabetes. But it’s not a problem when you eat acid couac. LS5032, Saint-Georges.

The nutrition and food transition, and some of its aspects, have been previously documented in connection with Parikwene food systems and diet (27, 30, 31, 61, 73). Amidst Parikwene narratives linking such transitions to increased consumption of imported and market foods with an increasing prevalence and incidence of diabetes (69), cassava tubers remained the staple food item in Parikwene households, though not unscathed from changes through time. The most notable change reported by Parikwene participants is the historically widespread practice of consuming cassava flatbread, which has now largely been replaced by the transformation of cassava tubers into couac. This shift was reported to have happened in the early 1940s; the consumption of cassava flatbread can now be characterized as an occasional practice, whereas couac is commonly observed in nearly every Parikwene household.

In relation to diabetes, couac and cassava tuber were discussed by participants in 93.3% of all interviews (70/75). Despite Parikwene narratives against the centrality of couac as a direct cause for diabetes, its consumption in dietary interventions for the management of diabetes was recognized, nonetheless. In 83.3% of interviews (40/48), Parikwene participants reported two principal types of couac that were involved in glycemic control, whereas this was only brought up by one non-Parikwene participant working as a home nurse. These were called sweet couac (*puveye kiteye*) and acidic couac (*puveye suweine*), whose names characterize their organoleptic properties. In 72.9% of interviews (35/48), Parikwene participants considered acidic couac to be better for diabetics over sweet couac. In three interviews, participants stated that it did not affect diabetes any differently from sweet couac whereas participants in two other interviews specified that it depended on the person, adding that their personal experience was different to what they have observed among other members of their community. The only non-Parikwene participant to be familiar with these different types of couac also noted that acidic couac did not increase glycemia as dramatically than sweet couac. Accordingly, many diabetics interviewed switched from sweet couac to acidic couac in an effort to keep their glycemic levels lower. One participant from Macouria, LS6022, explains: “There is couac that is very sweet. The sweet couac, it is not good for diabetes. If you eat it, it raises your diabetes. There is the couac that is not really sweet. If you eat it, it’s good.”

**Figure 5:**
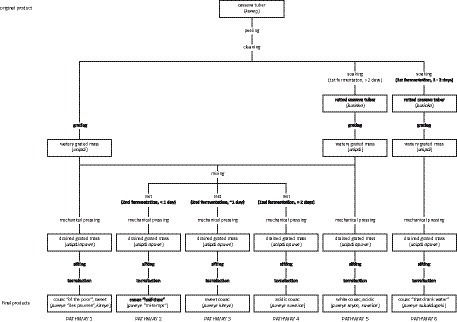
The operational sequence, or *chaîne opératoire*, of couac production.

**Figure 6:**
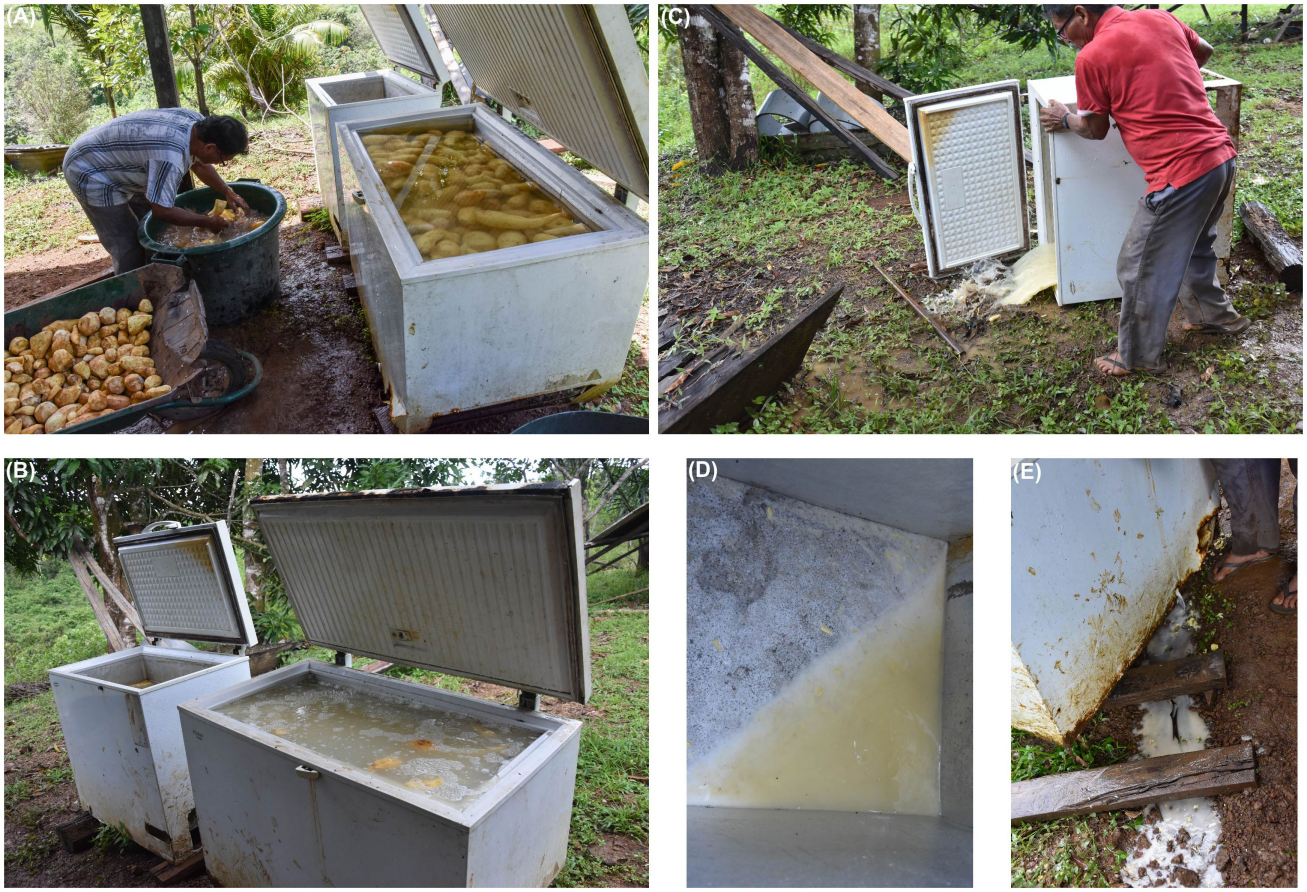
Illustrations of the retting process of cassava tubers (*Manihot esculenta*). Cassava tubers cleaned **(A)** and retting for 3 days **(B)**. The water used to ret the tubers is then discarded **(C)** along with a sizeable amount of starch, which can be observed in the bottom of the recipient **(D)** and on the ground **(E)**. Photo credits: M. Rapinski.

Slight variations to the operational sequence, or *chaîne opératoire*, of couac production led to the preparation of several varieties of couac based on the number of fermentation steps and their duration (Figure 5). Despite these multiple approaches, sweet and acidic couac derived from double fermentation were the principal types (pathway 3 and 4; Figure 5), as evidenced by narratives regarding their implication in glycemic control. These two differed from one another in the duration of the second fermentation, whereby acidic couac resulted from the mixed aqueous mass of retted and freshly grated tubers fermenting for 2 days or more (Figure 5). The number of days allowed for cassava tubers to macerate in water (1st fermentation) and rest (2nd fermentation) depends on the level of acidity desired in the end results. This may vary from a minimum of 2 days up to 1 week for a very acidic tasting couac. Due to its undesired organoleptic properties, a sizeable amount of starch is discarded following the maceration of tubers (Figure 6). Additionally, the process of grating fresh tubers allows for the optional step of extracting starch (*kayut*) for culinary purposes before the grated mass is left to ferment with the retted mass, thus further reducing the starch content in the end product. Although some Parikwene agroecological calendars report that the transformation of cassava tubers traditionally occurs in April (66) or October (67), the process of couac production was observed at any time of the year, providing that cassava tubers are sufficiently large to harvest. In fact, varieties with short (nine months) and more or less long (up to two years) maturity cycles have been found growing in Parikwene cassava fields, facilitating their harvest throughout the year (56). Quantities produced depend on the size of households, the number of people involved, the amount of time allocated to this activity and the purpose of production (i.e., personal or commercial). As an example, roughly 50 kg of couac were produced on the day of torrefaction during one of the visits to the cassava house. Couac is stored in waterproof containers, therefore rendered readily available for consumption at meal time. When the stock is depleted, it is replenished by repeating the fabrication process or purchased through short-circuit commerce in the Parikwene community.

Giving the number of variables involved in couac production, specific choices throughout the transformation process depend on know-how and personal preferences in taste. Some participants did not hesitate to experiment with the operational sequence in order to find a balance between taste and desired effect on diabetes. One participant from Saint-Georges explains:

For couac, we found the system […] There is an elder who told us: ‘Listen, you see, the person who has diabetes, he cannot eat sweet couac. Instead, go acidic.’ […] We do the acidic couac, and that… And that’s good. Like that, the person, they felt good with the acidic couac. And now, and afterwards, there are people who say: ‘No, I do not want to eat acidic couac anymore. I’m fed up with this.’ But what can we do? […] And then we found another way. They said: ‘No, that’s not how we are going to do it. We’re going to do neither sweet nor acidic. It will stay in the middle.’ You see? […] This is what we are doing now LS5003, Saint-Georges.

This mixture results in the couac produced from pathway 2 (Figure 5); due to participants reporting this as a relatively new innovation in couac production, it did not possess a specific name in Parikwaki. Indeed, it is organoleptic perception and properties that primarily come into play when distinguishing sweet from acidic couac. Despite the importance of technique and process in differentiating the two, couac derived from one or no fermentation steps could also be considered a type of acidic or sweet couac, respectively (pathway 1 and 5; Figure 5), but were also given other names due to diverging characteristics like texture and color. In general, these end products were generally not preferred, nor was their naming based on taste considered authentic with respect to savoir-faire in couac production. Notwithstanding, changes to the operational sequence of couac production appears to be an ongoing process.

Given the shift from cassava flatbread consumption to couac occurring during the mid-20th century, documented changes to the transformation of cassava tubers have since continued to provide different variants of couac (57, 88). Parikwene narratives clearly linked the adoption of acidic couac with the recognition of diabetes as a populational health problem, and this process can be inductively reasoned to have started gradually by the early 2010s. Informative posters displayed at a communal shelter built *circa* 2015 for couac production in Saint-Georges describe the double fermentation process of couac, but for a period of 24 h during the second fermentation (pathway 3; Figure 5), thereby producing sweet couac. A detailed description of the operational sequence of couac production among French Guianese Creole People has cited the first stage of fermentation consisting of soaking the tubers for 24–48 h (pathway 6; Figure 5) before grating, or grating the fresh tubers immediately after they have been cleaned and rinsed (pathway 1; Figure 5) (88). Jolivet (89) notes that these methods are rarer than couac made from a double fermentation process akin to pathways 2–4 (Figure 5), adding that tubers are more commonly soaked for 3 days then mixed with freshly grated tubers for a minimum of 12 h (pathway 2; Figure 5). Among Parikwene People, Boutefeu et al. (57) report a single fermentation step of soaking the tubers for a few days as an occasional step between peeling and grating (pathway 5; Figure 5). Both Bereau et al. (88) and Boutefeu et al. (57) make no mention of a double fermentation. Lecointe (62), on the other hand, describes three different pathways employed by Indigenous Peoples to process bitter cassava tubers in Brazil. The first which results in *farinha d’agua* requires soaking the tubers in water for four to 5 days before grating (pathway 5, Figure 5), the second which results in *farinha secca* requires the soaking tubers to be removed from the water before they become soft (pathway 6; Figure 5), and the third which results in *farinha suruhy*, the tubers are grated without soaking (pathway 1; Figure 5). Whereas the first is said to be almost exclusively used in the Brazilian Amazon, the two others are commonly used in Brazil’s southern states (62).

### 3.4. More on the evolution of couac

The adaptation of couac production in the management of diabetes is grounded in a dynamic Parikwene knowledge system followed with a keen sense of observation. In integrating diabetes to their knowledge system, Parikwene have associated the illness with the following characteristic: (1) sweetness, due to its link with sugar and sweet foods, and (2) heat, due to explanations given to certain symptoms like the evacuation of fluids through polyuria and diarrhea as a consequence of the body overheating and needing to be cooled by replenishing with water, hence polydipsia (69). These sweet and hot characteristics are in keeping with Parikwene medicines that are frequently described based on their organoleptic (i.e., sour and bitter tasting) and caloric (i.e., cold, cool and refreshing) properties (69).

The notion of hot and cold that underlines the description of diabetes and medicines is similar to the local Creole medicinal system (70, 90); three centuries of close proximity between Creole and Parikwene populations is undoubtedly important in exchanging knowledge between the two. Nonetheless, the hot and sweet attributes given to diabetes are important factors driving current attitudes toward the illness and its management as these inadvertently bridge together biomedical and contemporary Parikwene concepts. Sweetness connects sugar, the main biomedical measure and target of diabetes treatments, to a large repertoire of Parikwene treatments characterized by their organoleptic properties, such as acidity for treating hot illnesses. Furthermore, these sensory characteristics bridge medicines and food (69), of which sweet and acidic couac are a perfect example.

Choices regarding which type of couac to consume for the management of diabetes are adopted on a personal basis. These choices are notably influenced by the experience of adverse reactions following the consumption of a specific type of couac, or other food items. The symptoms reported by diabetic participants include:

- numbness in the mouth;
- dry mouth;
- sudden fatigue and weakness;
- perspiration;
- increase in body heat;
- diarrhea;
- polyuria.

Blood glucose levels were often measured with a glucometer during such events, either personally or assisted by a family member or nurse, to confirm whether there is hyperglycemia. Paying attention to body signals, symptoms of diabetes, and to glucometer readings were the primary practices in self-guiding dietary interventions around diabetes. One participant explains how the glucometer helps in confirming the effects of acidic couac on diabetes:

Sweet couac raises diabetes. The doctor takes blood, he puts it in a device, he looks, it goes up a lot. If it goes up to 5 or 6 grams, it’s too high, it’s a lot. If it’s 1 or 1.60 or 2 and 2.80 grams, it’s good, it’s not that high. We see the difference when we eat sweet couac and acidic couac, we see the difference in the device. LS5032, Saint-Georges.

Changes in the organoleptic properties of acidic couac likely result from biochemical changes that occur during the soaking and fermentation of cassava tubers. Such changes are well-documented for a variety of derived cassava products requiring soaking and fermentation where fungi (i.e., *Candida* spp.) and bacteria (i.e., *Bacillus* spp., *Lactobacillus* spp., *Klebsiella* spp., *Leuconostoc* spp., *Corynebacterium* spp.) have been found to break down starches and sugars into organic acids (91, 92). This not only results in the acidification of the tubers and the derived foodstuff, but also a decrease in sugar and starch content (91–95), key components in lowering the GIs of foods (96).

However, soaking and fermentation are just two of a multitude of steps that are used in various combinations to produce a variety of products from cassava tubers (91, 94, 97). One study found that various methods for transforming these tubers, including soaking and fermentation, did not affect the GIs of four of its food derivatives (98). A recent study, however, found that processing differences between two cassava-derived foods (i.e., fufu and gari) distinctly impacted their content in resistant starch and rapidly digestible starch, both of which release glucose at different rates (99). Despite this, different cassava derived foodstuff were generally found to possess high GIs (96, 98). Moorthy and Mathew (91, p. 73) note that “glaring inconsistencies […] in some of the results reflect the differences and variations in the artisanal processes followed in the preparation of these products.” This is not taking into account that the starch content of *M. esculenta*, genetically diverse worldwide (56, 100), is highly variable from one variety to the next (101). This is all the truer for acidic couac where, to the best of our knowledge, there is a lack of data regarding its GI and whose preparation is conditional on personal preferences in taste and purpose, e.g., personal consumption, variety, sale, extraction of tapioca. Whether or not acidic couac and its various methods of preparation lead to lower GIs should be the focus of further in-depth studies. However, anecdotal evidence provided by participants through glucometer readings and the expression of symptoms of hyperglycemia (i.e., dry mouth, weakness, polyuria, and diarrhea) lend support to this hypothesis.

## 4. Conclusion and final considerations

The consequences of a narrow and constrained discussion on dietary choices are recognized by healthcare professionals who acknowledge the lack of relevant information and the difficulty around communicating about certain foods like couac, or cassava consumption. The source of a variety of food products in the Parikwene food system, *M. esculenta* can be considered a cultural keystone species with strong links to the land through cultural keystone places, namely swidden-fallow fields. At such places, couac production at the “cassava house” facilitates the transmission and exchange of know-how and stories, thus preserving language as well as strengthening family and community ties. Undeniably the principal source of dietary subsistence, *M. esculenta* is also an important economic driver to some, and through the production of basketry both sold and used in cassava transformation, a driver of material culture for many more (45). Hence, remarks regarding the implications of this food item in diabetes are delicate, striking directly at characteristics that define Parikwene identity and way of life. In fact, this is at the core of a struggle against being seen as culprits in perceived allegations stemming from the healthcare system regarding the incidence of diabetes. Unbeknown to participants connected to the healthcare sector, however, one of the most notable antidiabetic practices adopted by the Parikwene of Saint-Georges and Macouria is the consumption of acidic couac for better glycemic control. Indeed, when Boutefeu et al. (57) documented the varietal diversity of *M. esculenta* and the operational sequence of cassava flour in 2002, it was not that of acidic couac which they recorded.

Attitudes and practices around dietary interventions of diabetes show us how aspects of biomedical knowledge of the disease have not only been integrated into the Parikwene knowledge system, but have also merged with authentically Parikwene concepts. This translates into grassroots practices that make use of biomedical tools like glucometers to guide dietary choices around cassava consumption. Described more generally in local malaria treatment practices in eastern French Guiana (102), such mixing of biomedicine with Parikwene medicine is not uncommon and points to a desire for autonomy in the management of diabetes. As argued by Hobsbawm and Ranger (103), there is a constant invention of tradition; far from being passive, this highlights how Parikwene are eminently dynamic in their relationship to illnesses and adapt to the strong changes they have been experiencing for several decades. Although at-home visits from nurses are important in this process of self-management of diabetes, the lack of adapted information regarding the nutritive qualities and potentiating effect of couac consumption on diabetes that is available to healthcare professionals means that they have to rely strongly on these self-assessment measures. In the absence of proper information, patient education programs, like the one offered to HIV patients in Saint-Georges (104, 105), may be developed to harness peoples’ desire for self-management by focusing on the use of glucometers and the recognition of distinctions between symptoms of hyperglycemia and hypoglycemia. In the meantime, research to further understand the implication of cassava tuber transformation on glycemic control is pertinent. The ethnomedicinal hypothesis that acidic couac has a better impact on glycemic control can be tested by determining their GIs following different transformation methods. This can be followed by microbiological studies like high-throughput DNA and RNA sequencing to assess the microbiome diversity at each fermentation step (106, 107), methods that could also be used to understand differences among cassava varieties, as well as phytochemical studies to assess the elimination of toxic cyanogens with each processing step.

## Data availability statement

The datasets presented in this article are not readily available because audio recordings and transcriptions are kept confidential to protect the identity of participants. Requests to access the datasets should be directed to MR, michael.rapinski@umontreal.ca.

## Ethics statement

The studies involving human participants were reviewed and approved by Comité d’éthique de la recherche en arts et en sciences of the Université de Montréal (CERAS-2016-17-081-P). The participants provided their written informed consent to participate in this study. Written informed consent was obtained from the individual(s) for the publication of any potentially identifiable images or data included in this article.

## Author contributions

MR conducted this study in the context of his doctoral thesis. AC and DD supervised the work as doctoral advisors. All authors contributed to the manuscript revision, read, and approved the submitted version.

## Funding

Financial support was provided by a PICS (N° 07779 “*Gestions des territoires, des ressources et systèmes de santé autochtones comparés* (*Guyane française-Québec*)”) from France’s CNRS to AC and DD, the OHM Oyapock, Labex DRIIHM (ANR-11-LABX-0010) and Labex CEBA (ANR-10-LABX-25-01), as well as a CIHR Doctoral Research Award (201510DAR-358297-196992) and a travel award from the Université de Montréal to MR.

## Conflict of interest

The authors declare that the research was conducted in the absence of any commercial or financial relationships that could be construed as a potential conflict of interest.

## Publisher’s note

All claims expressed in this article are solely those of the authors and do not necessarily represent those of their affiliated organizations, or those of the publisher, the editors and the reviewers. Any product that may be evaluated in this article, or claim that may be made by its manufacturer, is not guaranteed or endorsed by the publisher.
